# Inhibitory and excitatory conditioned cues associated with alcohol differentially modulate the nucleus accumbens shell: involvement of 5-HT_7_ receptors

**DOI:** 10.3389/fpsyt.2025.1634350

**Published:** 2025-09-03

**Authors:** Sheketha R. Hauser, Gerald A. Deehan, Christopher P. Knight, Robert A. Waeiss, Eric A. Engleman, Phillip L. Johnson, William J. McBride, Richard L. Bell, William A. Truitt, Zachary A. Rodd

**Affiliations:** ^1^ Department of Psychiatry, Indiana University School of Medicine, Indianapolis, IN, United States; ^2^ Stark Neurosciences Research Institute, Indiana University School of Medicine, Indianapolis, IN, United States; ^3^ Department of Psychology, East Tennessee State University, Johnson City, TN, United States; ^4^ Department of Anatomy, Cell Biology and Physiology, Indiana University School of Medicine, Indianapolis, IN, United States

**Keywords:** alcohol use disorders (AUD), alcohol-seeking behavior, dopamine, glutamate, serotonin, dopamine receptor (D1), serotonin receptor (5-HT7), alcohol preferring (P) rats

## Abstract

**Introduction:**

The ability of conditioned cues to evoke drug craving is considered a critical factor precipitating relapse of drug use. The nucleus accumbens shell (AcbSh) is a structure that mediates drug-seeking via the influence of associations formed between conditioned cues and drug reward.

**Methods:**

In the present experiments, alcohol-preferring (P) rats were exposed to three conditioned odor cues; CS+ associated with alcohol self-administration, CS− associated with the absence of alcohol (extinction training), and a neutral stimulus (CS^0^) presented in neutral environment with no association to alcohol. The experiments examined the effects of the conditioned cues on extracellular levels of dopamine (DA), serotonin (5-HT), and glutamate (GLU), as well as the pattern of activation of D1 receptor-containing neurons in the AcbSh. The involvement of 5-HT_7_ receptors within the AcbSh in regulating context- and cue-induced alcohol-seeking was also determined.

**Results:**

Presentation of the CS+ resulted in increased extracellular DA levels and reduced 5-HT levels in the AcbSh, as well as increased activation of D1 receptor-containing neurons. In contrast, presentation of the CS− decreased extracellular DA and GLU levels in the AcbSh. The conditioned cues did not affect DA levels in the Acb core. The intra-AcbSh administration of a 5-HT_7_ antagonist enhanced context- and cue-induced alcohol seeking, whereas a 5-HT_7_ agonist reduced these behaviors.

**Discussion:**

Overall, the data suggest that there are distinct neurocircuits within the AcbSh that mediating the effects of excitatory and inhibitory conditioned cues on motivated behavior. While this work highlights a complex interaction of several neurotransmitter systems, it may also suggest a potential role for behavioral therapies involving extinction training and 5-HT_7_ receptor activation as potential targets for the treatment of cue-induced drug-seeking behavior.

## Introduction

The nucleus accumbens (Acb) is a key neuroanatomical region involved in drug reward and drug-seeking behaviors. It comprises the shell (AcbSh) and the core (AcbC), both of which are associated with contextual and cue-induced alcohol-seeking behaviors (1–5). The AcbSh is pivotal for drug reward as repeated, response-based exposure to addictive substances enhances dopamine (DA) transmission, thereby strengthening the associations between stimuli and drugs (6). Preclinical imaging research has demonstrated that both AcbSh and AcbC respond to alcohol-related excitatory conditioned cues (CS+) (7, 8). Furthermore, inactivating AcbSh and AcbC can reduce CS+-triggered alcohol-seeking behavior in the renewal model (3). Additionally, inhibiting AcbSh with GABAB (baclofen) and GABAA (muscimol) agonist solution disrupts cue-induced behaviors in both the original alcohol training context and a new setting, with no impact on a neutral context (9).

The ability of excitatory conditioned (CS+) cues to induce an intrinsic motivational state of drug craving is thought to contribute to the high relapse rate in alcohol use disorders (AUDs) and drug addiction (10, 11). Clinical data indicate that CS+ cues can enhance self-reported craving for alcohol while concurrently reducing behavioral inhibition and cognitive performance (12, 13). Stimuli associated with the availability of alcohol (CS+) can augment context-induced alcohol-seeking (14) and can be observed in the Pavlovian spontaneous recovery (PSR) (15, 16) and renewal (17) models of context-induced alcohol-seeking.

On the other hand, stimuli can also be paired with the absence of a reinforcer (inhibitory conditioned cues; CS−), thereby signaling the unavailability of a potential reward, theoretically leading to a decrease in stimulus-induced behavior. Research has demonstrated that a CS− can decrease alcohol seeking in the ABA renewal model [i.e., alcohol consumption environment (context A), extinction training (context B), and renewal context seeking (A)] when presented in all contexts (18, 19) or paired with only extinction to the context that was originally associated with alcohol administration (extinction cues) (20, 21). Such inhibitory conditioning with cues is not comparable to conditioned inhibition (a cue paired with an aversive event used to reduce the expression of unrelated behaviors) because the presentation of a CS− activates a distinct neurocircuitry compared to conditioned inhibition (22). Presentation of a CS− can reduce alcohol seeking in outbred rats trained to self-administer beer (20, 21) as well as in P rats (5, 15, 16). Furthermore, a CS− associated with cocaine was able to reduce CS+-induced cocaine seeking when cues were presented simultaneously (9, 23). This data would suggest that the CS+ and CS− activate neurocircuitry that acts in a push/pull fashion with regard to eliciting the appropriate behavior given the cue.

The three major neurochemical systems focused on herein (DA, glutamate: GLU, and serotonin: 5-HT) have all been found to play a role in the manifestation of cue-induced drug-seeking behavior. The manner in which each system contributes to the process of learning associations between cues predictive of alcohol access, whether they are contextual or conditioned cues, is complex and dependent on the function of distinct neurocircuitry to either drive or inhibit behavior. Alterations in DA transmission prior to exposure to a reward have been hypothesized to set expectations for reward likelihood in the future (24). The presentation of a CS+ can increase DA neuronal activity, while the presentation of a CS− can suppress DA neuronal activity (25). Additional research has identified the activity of DA, specifically at the D1 receptor, as a key component mediating excitatory drug seeking as both systemic (18, 19, 26), and intra-Acb (18, 19) antagonism of D1 receptors can attenuate CS+ induced alcohol-seeking behavior without a commensurate effect on CS−-associated behavior. Taken together, these studies would suggest that the cue-induced behavior elicited by exposure to a CS+ or a CS− occurs via different signaling afferent profiles within the Acb which ultimately alters activity in structures downstream of the Acb DA system and additional neurochemical systems.

Alterations of brain GLU function, specifically GLU pathways that originate in the prefrontal cortex and project to the Acb, have been linked to the actions/motivational properties of alcohol (27, 28)—for example, increased extracellular concentrations of GLU have been reported in the AcbSh of P rats following free-choice alcohol drinking (29). In addition, pharmacological studies have demonstrated that targeting metabotropic GLU receptors (i.e., mGlu5 antagonism or mGlu2/3 agonism) systemically reduced the cue-induced (30) and PSR-associated expression of alcohol-seeking behavior (31) in P rats. Moreover, the selective inhibition of mGlu5 within the infralimbic cortex (IFL) produced a greater reduction in cue-induced alcohol seeking compared to the same treatment in the prelimbic cortex (PrL), further illustrating the complex nature of glutamatergic influence on the Acb (32). Nevertheless, the impact of conditioned cues on GLU transmission in the AcbSh requires further attention.

Loss of control is a major feature of AUDs, and deficits in 5-HT may contribute to behavioral disinhibition, thereby increasing the risk of relapse (33). Contextual and drug-related cues have been shown to increase behavioral disinhibition (34). Antagonism of different subtypes of 5-HT receptors and drugs that increase 5-HT neurotransmission can reduce CS+-induced drug-seeking behaviors (35–38). However, a limited number of studies have examined 5-HT involvement in mediating cue- and context-induced alcohol-seeking behaviors. One of the most recently discovered 5-HT receptor subtypes is the 5-HT_7_ receptor, which is expressed in mesocorticolimbic areas, including the Acb. In addition, 5-HT_7_ receptors can modulate DA neuronal activity within the VTA (39). It has been suggested that 5-HT_7_ receptors play an important role in reward devaluation processes (40) and 5-HT_7_ receptors can attenuate drug withdrawal symptoms (41). Collectively, these findings make the 5-HT_7_ receptor a novel target to examine its involvement in alcohol-seeking behaviors as well as determine if 5-HT_7_ receptors within Acb are involved in mediating alcohol-seeking behaviors.

The objectives of the current study were to determine the underlying effect of conditioned odor cues on neurochemical efflux within the Acb and the subsequent role of DA and 5-HT signaling on cue-induced alcohol seeking. We first hypothesized that the presentation of the CS+ (associated with alcohol access) and CS− (associated with the absence of alcohol) would have distinct effects on DA, 5-HT, and GLU transmission in AcbSh. It was further hypothesized that only CS+ would increase neurons possessing the D1 receptor in the AcbSh, and the manipulation of 5-HT_7_ receptor-containing neurons within the AcbSh would produce distinct alterations in alcohol-seeking behavior associated with excitatory versus inhibitory conditioned cues as well as contextual cues associated with alcohol access.

## Materials and methods

### Animals

Adult female P rats weighing 250–325g at the start of the experiment were used. The rats were maintained on a 12-h reversed light–dark cycle (lights off at 0900 h). Food (Teklad 2918X; Envigo, Indianapolis, IN, USA) and water were available in the home cage ad libitum throughout the experiment. All animals were double-housed in shoebox cages, except for those that underwent surgery, which were single-housed in the shoebox cages after surgery. All research protocols were approved by the Institutional Animal Care and Use Committee and are in accordance with the guidelines of the Institutional Care and Use Committee of the National Institute on Drug Abuse, National Institutes of Health, and the Guide for the Care and Use of Laboratory Animals (42).

### Alcohol self-administration and cue conditioning

Details of the operant chambers have been previously described (5, 15, 16, 43). Water and 15% (v/v) alcohol were concurrently available in standard two-lever operant chambers. Upon a reinforced response, the rats were given access to 0.1 mL of reinforcer for 4 s presented in a 0.1-mL dipper cup. The operant sessions were 60 min in duration and conducted daily. The P rats were allowed to self-administer alcohol and water for 10 consecutive weeks. During this period, a conditioned odor cue (CS+; excitatory odor stimulus) was present for all 70 sessions. The average alcohol intake of all rats was approximately 1.2 g/kg/session. Past research indicated that this level of intake would result in blood ethanol levels of 60–80 mg% (44). The end work requirement was a fixed ratio 5 (FR5) for alcohol and an FR1 for water. Exposure to a neutral odor stimulus (CS^0^) began on session 56 (i.e., week 9 to 10 of alcohol self-administration) and was continued until session 77 (i.e., week 11 of extinction sessions). Exposure to the neutral stimulus occurred in a unique, non-drug-paired environment for 1 h and occurred daily before the operant session. Following the 70 sessions of alcohol self-administration/CS+ exposure, all rats received seven sessions of extinction training (no water or alcohol available). During extinction training, another odor cue (CS−; inhibitory odor stimulus) was present throughout the sessions.

Odor cues were counterbalanced across subjects. The conditioned odor cues included peppermint, almond, and banana, and each odor was used as the CS+, CS−, or CS^0^ in separate groups of rats. All rats were conditioned to CS+, CS−, and CS^0^ but were only exposed to one of the conditioned stimuli for the PSR test. During conditioning, odor cues for CS+ and CS− were emitted from a treated cotton ball placed outside the operant chamber but within the sound attenuation cubicle.

**Figure 1:**
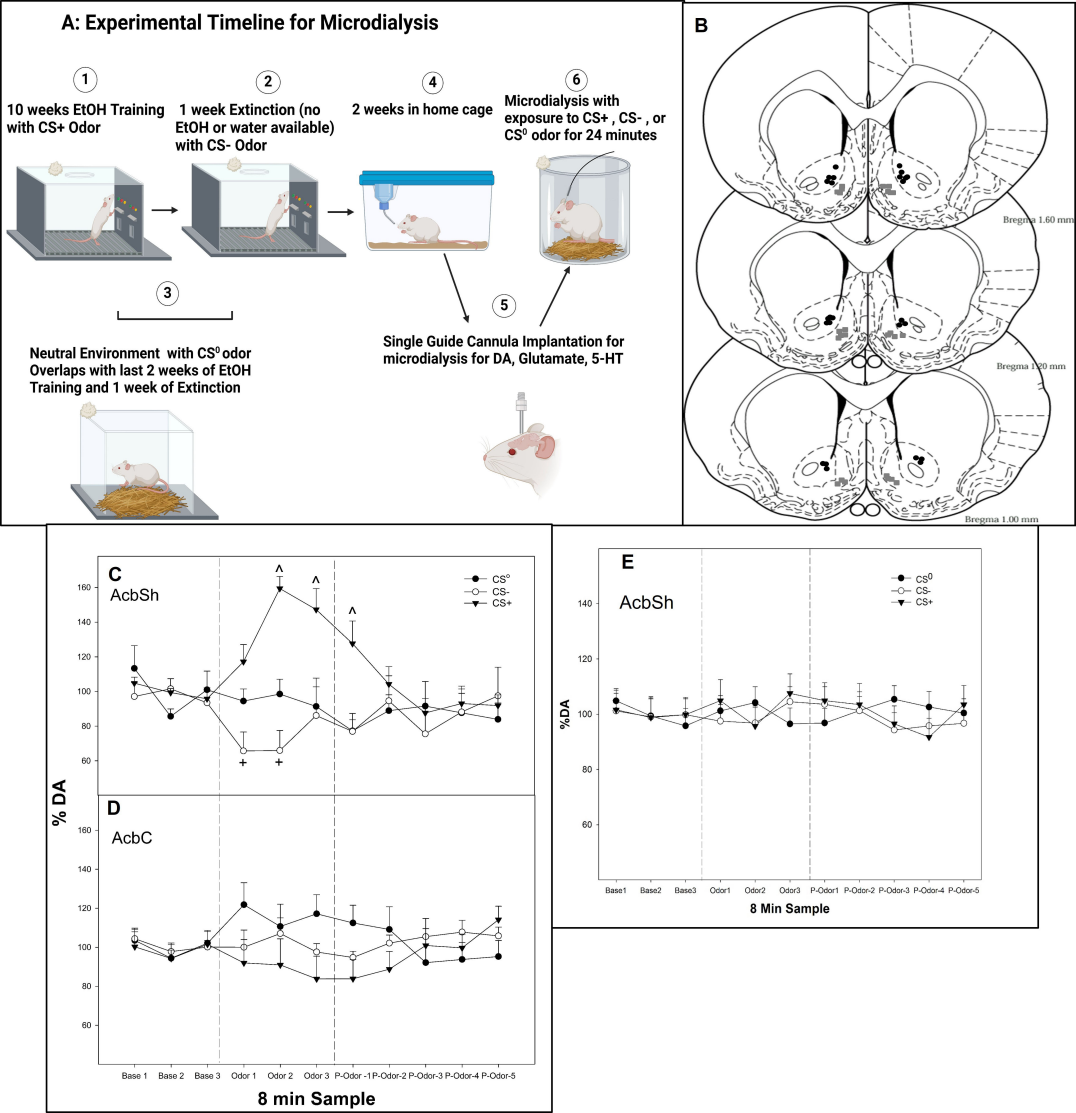
**(A)** Experimental timeline for microdialysis (created with BioRender.com). **(B)** Representative placements of microdialysis probes and microinjections in the AcbSh or AcbC experiments 3–6. The gray squares represent sites in AcbSh, and the black circles represent sites within AcbC. **(C)** Mean (± SEM) change in extracellular DA levels (nM) as % of baseline in the AcbSh and **(D)** mean (± SEM) change in extracellular DA levels (nM) as % of baseline in the AcbC in P rats exposed to conditioned cues (CS^0^, CS+, and CS−) in a non-drug-paired environment for 24 min. **(E)** Mean (± SEM) change in extracellular DA levels (nM) as % of baseline in the AcbSh in P rats exposed to **“**mock**”** conditioned cues (CS^0^, CS+, and CS−) in a non-drug-paired environment for 24 min. The rats were never exposed to alcohol, and conditioned cues were all paired with water in the operant chamber. The rats experienced the identical experimental protocol depicted in image 1 but with no alcohol. ^indicates that the DA levels in the AcbSh are elevated in the rats exposed to the CS+ compared to all other groups and baseline levels. + indicates that the DA levels in the AcbSh are lower in the rats exposed to the CS− compared to all other groups and baseline levels. Base, baseline prior to exposure of odor; Odor, the effects of CS odors; P-odor, effects after the removal of the odor.

After extinction training, all rats were maintained in the home cages for 14 days. The effects of conditioned cues on alcohol-seeking were tested in the PSR paradigm for at least four sessions. Lever contingencies and dipper functioning were maintained, but alcohol and water were absent. **Figure 1** provides a timeline (created with BioRender.com) for the experimental protocol. A thorough discussion about the length of cue presentation has been discussed elsewhere (5, 15, 16). Given the olfactory nature of the cues and the necessity to maintain the appropriate cue exposure during training and testing, the laboratory personnel were not completely blind to the group assignments. There were a limited number of laboratory personnel on staff who trained and tested the animals, and the cues were presented in a unique but serial manner (i.e., CS+ followed by CS−; CS^0^ in separate context). Thus, it would have been impossible to maintain a completely blind experimental design. It is important to note, however, that all animals were randomly assigned to their testing (olfactory cue exposure, no alcohol) group following training.

#### Experiment 1: determine alterations in extracellular DA and 5-HT levels in the AcbSh or AcbC following exposure to conditioned cues

At 1 week after the last extinction training session, all rats were implanted with an 18-gauge guide cannula (Plastic One, Roanoke, VA, USA) aimed at the AcbSh (+1.7 mm AP, +2.4 mm lateral to the midline, and 5.4 mm ventral from the surface of the skull at a 10° angle) or AcbC (+1.7 mm AP, +2.4 mm lateral to the midline, and 4.4 mm ventral from the surface of the skull at a 10° angle; **Figure 1B**) while under isoflurane anesthesia [2%] (45). The guide cannula was aimed at 2.0 mm above the target region. The analgesics administered to the animals were bupivacaine hydrochloride 0.5% (1 mg/kg, administered once during surgery) and carprofen (10 mg/kg, administered subcutaneously, once during surgery and 2 days postoperatively). From the fourth to the seventh recovery day, the animals were handled for at least 5 min and habituated to microdialysis chambers each day (**Figure 1A**, microdialysis timeline; Created in BioRender. Hauser, S. (2025) https://BioRender.com/uidm7kn). On the final recovery day, loop style microdialysis probes (active length 2.0 mm, Spectra/Por RC, inner diameter 200 μm, molecular weight cutoff: 13,000; Spectrum Laboratories Inc., Rancho Dominguez, CA, USA) were inserted into the AcbSh or AcbC following handling/habituation.

Standard microdialysis procedures occurred in a second non-drug paired environment. A liquid swivel was used to connect the probe to the microinfusion pump [for more details, see (5, 45, 46)]. Artificial cerebrospinal fluid (aCSF) was perfused through the probe at 1.0 µL/min with a syringe pump (aCSF composition in mM: NaCl, 145; KCl, 2.7; MgCl_2_ 1.0; CaCl_2_, 1.2; pH 7.4) for a 90-min washout period. Four to five baseline samples were collected at 8-min intervals. The rats were randomly assigned to groups exposed to CS^0^, CS−, or CS+ for 24 min (three sample periods; *n* = 6–10/group). After removing the odor cue, five to six additional samples were collected. Dialysate samples were collected in vials containing 5 µL of preservative for DA or 5-HT (0.2 mM ethylenediamine tetraacetic acid, 0.33 mM L-cysteine, and 0.05 mM L-ascorbic acid in 0.1 N acetic acid) immediately frozen on dry ice and stored at −70°C until analysis. All samples were analyzed by using high-pressure liquid chromatography with electrochemical detection (HPLC-ED) calibrated for monoamine detection as previously described (5, 45, 46).

As an additional control, the experiment was replicated in rats that only had water available in the operant chamber but were exposed to all conditioned cues. The rats were treated identically as alcohol rats and were randomly assigned to the mock CS^0^, CS−, or CS+ for 24 min (*n* = 4/group). Using the same approach as that used for the operant experiments, the laboratory personnel were not completely blind to the group assignments for the microdialysis studies. Following the operant olfactory cue associative training, the animals were randomly assigned to their testing (olfactory cue exposure, no alcohol) group. It was necessary for the research personnel to have knowledge of the group assignment to ensure proper collection, organization, and analysis of dialysate samples.

At the conclusion of the experiments, a 1% solution of bromphenol blue in aCSF was perfused through the probe to verify placement in the AcbSh. Brains were stored at −70°C until sectioned using a cryostat microtome. The histological placements were verified using the rat atlas (47), and animals with probe placements outside the AcbSh or AcbC were excluded from further analysis.

#### Experiment 2: determine alterations in extracellular GLU levels in the AcbSh following exposure to conditioned cues

**Figure 2:**
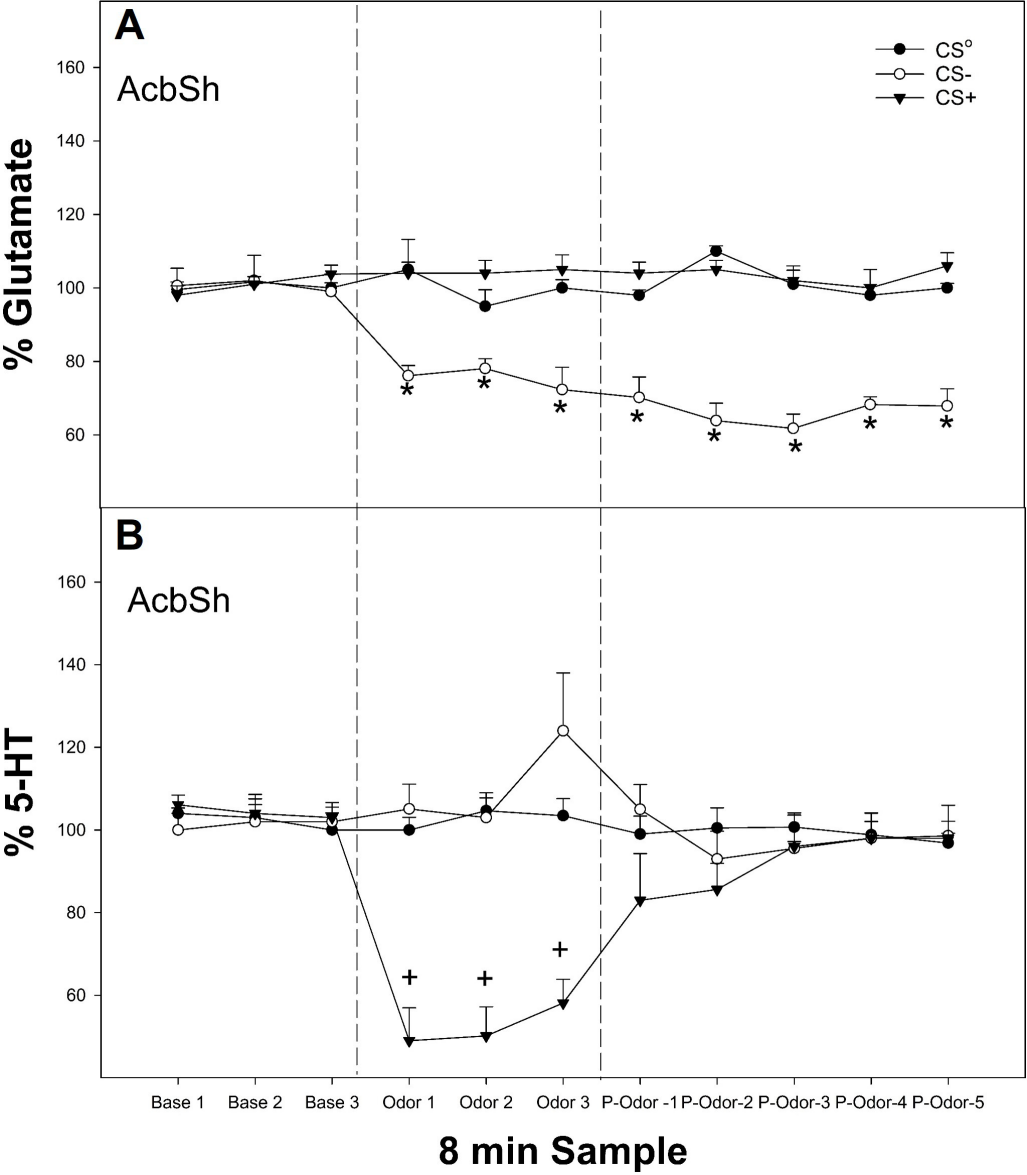
**(A)** Mean (± SEM) change in extracellular GLU (top panel) or **(B)** mean (± SEM) extracellular 5-HT levels in the AcbSh in P rats exposed to conditioned cues (CS^0^, CS+, and CS−) in a non-drug-paired environment for 24 min. *indicates the amount of GLU levels (μM) as % baseline in the AcbSh was decreased in the rats exposed to the CS− compared to all other groups and baseline levels. +indicates 5-HT levels (nM) as % of baseline in the AcbSh was lower in rats exposed to the CS+ compared to all other groups and baseline levels. Base, baseline prior to exposure of odor; Odor, the effects of CS odors; P-odor, effects after the removal of the odor.

For this experiment, microdialysis was conducted identically to experiment 1, except that the GLU levels (**Figure 2A**) were determined in the AcbSh (29, 46).

#### Experiment 3: dual labeling cFos and D1 receptors in the AcbSh following cue presentation

**Figure 3:**
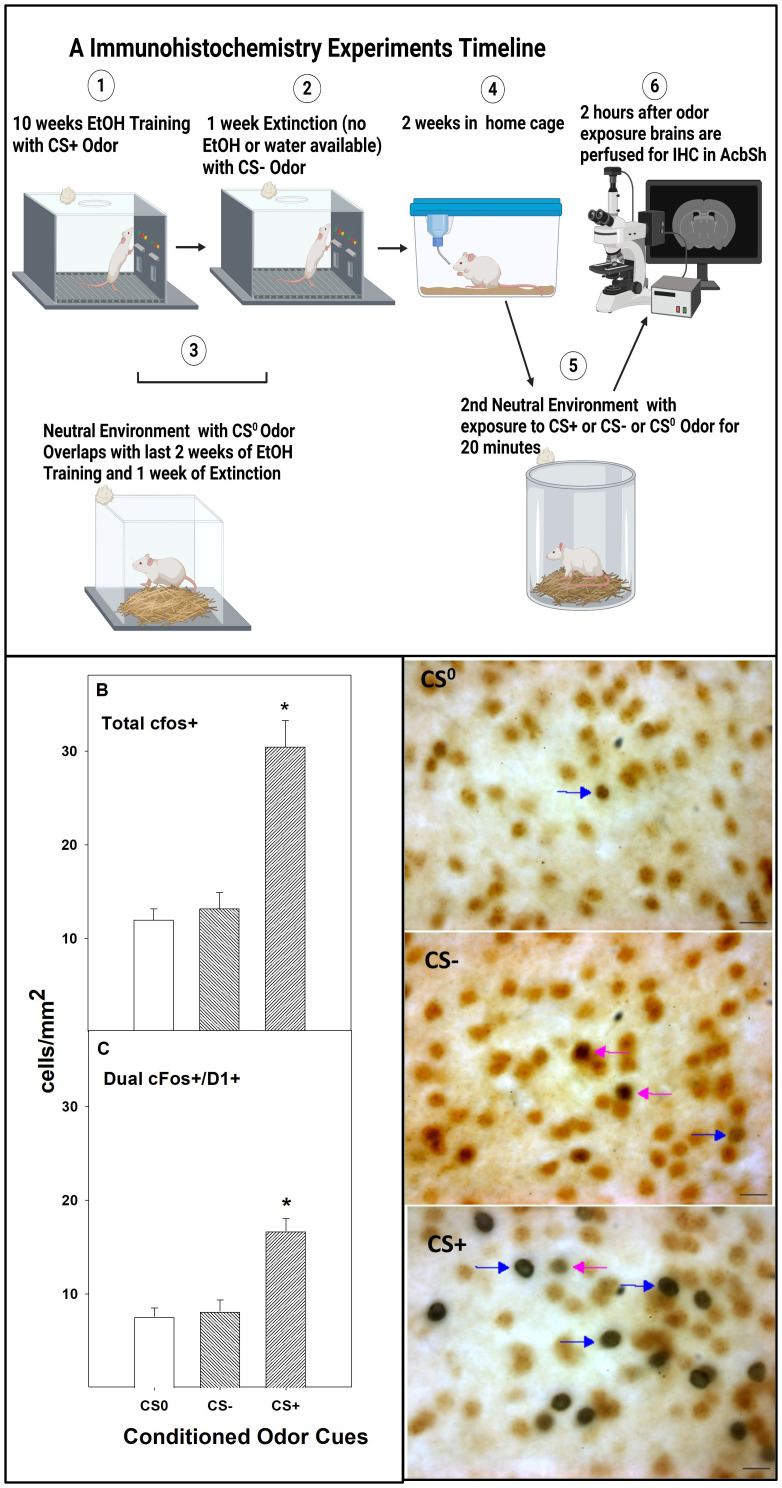
**(A)** Experimental timeline (created with BioRender.com). The mean (± SEM) cfos+ **(B)** and dual labeled (cfos+/D1+; **C**) following the presentation of conditioned cues in a non-drug paired environment. *indicates that rats exposed to the CS+ have significantly more cfos+ and cfos+/D1+ cells in the AcbSh than rats exposed to the CS^0^ or CS−. The images next to **(A, B)** are representative images of cfos and D1 staining in the AcbSh of rats exposed to CS^0^, CS−, or CS+. The blue right-pointing arrows indicate examples of single-labeled cfos cells, and the magenta left-pointing arrows indicate examples of dual-labeled cfos+ and D1+ cells. Scale bars, 100 µm.

Following the 14-day home-cage period, the rats were exposed to CS^0^, CS−, or CS+ for 20 min (*n* = 5–7/group) in the non-drug paired (neutral) environment; 2 h later, the rats were anesthetized (100 mg/kg sodium pentobarbital i.p.) and perfused with chilled isotonic saline followed by 4% paraformaldehyde in phosphate buffer (**Figure 3A** immunohistochemistry experimental timeline, Created in BioRender. Hauser, S. (2025) https://BioRender.com/r25v462). The brains were removed and processed using standard procedures (5, 7). The benefits of examining neuronal activity using the current protocol included the following (1): the rats were not allowed to actively express drug-seeking behaviors; thus, the effects of the cues were divorced from the performance of behaviors previously associated with the delivery of alcohol and (2) the rats were not exposed to the drug-paired environment, thus eliminating contextual-induced neuronal activity associated with drug-seeking.

A standard procedure was used to determine cFos immunohistochemistry (5, 7, 48–50). For cFos-only cell counts, AcbSh sections were rinsed in 0.1 M PBS (3 h) and then blocked in 1% hydrogen peroxide in PBS (10 min). After the PBS rinse, the sections were incubated in PBS with 0.1% bovine serum albumin and 0.4% Triton-X and then primary c-Fos rabbit antibody diluted 1:16k (cat. no. PC38 Calbiochem, San Diego, CA, USA) in PBS containing 0.1% bovine serum albumin and 0.4% Triton-X (16 h). The sections were then PBS-rinsed and incubated in biotinylated secondary goat anti-rabbit antibody (Vector Laboratories, Burlingame, CA, USA) diluted 1:500 in PBS containing 0.1% bovine serum albumin and 0.4% Triton-X (1 h). Following the PBS rinse, the sections were incubated in avidin–biotin–peroxidase complex (ABC Elite, Vector Laboratories, Burlingame, CA, USA) diluted 1:1,000 in PBS. After PBS rinsing, the sections were reacted with 0.02% 3,3′-diaminobenzidine (DAB; cat. no. D-5637, Sigma-Aldrich, St. Louis, MO, USA) enhanced with 0.08% nickel (II) sulfate and 0.012% hydrogen peroxide in PBS. Finally, the sections were rinsed (0.1 M phosphate buffer) and mounted; the slides were counterstained with neutral red, dehydrated, and cover-slipped. The number of c-Fos-immunoreactive cells per section was determined by two independent observers blind to the treatment groups, and four to seven sections per brain were analyzed.

The Acb sections used for dual labeling (i.e., staining cFos-D1 receptors in the Acb) underwent an additional overnight incubation with the appropriate primary antibody. After the overnight incubation with rabbit anti-c-Fos, the sections were then PBS-rinsed and incubated in biotinylated secondary goat anti-rabbit antibody (Vector Laboratories, Burlingame, CA, USA) diluted 1:500 in PBS containing 0.1% bovine serum albumin and 0.4% Triton-X (1 h). Following the PBS rinse, the sections were incubated for 1 h in ABC Elite diluted 1:1,000 in PBS. Then, it was incubated for 10 min chromogen reaction with SG Kit, washed for 30 min in PBS, and incubated with PBST for 1 h. Following the PBST incubation, the sections were incubated overnight with anti-rabbit D1 antibody (Alamone Lab, cat. no. ADR-001; used at 1:3,200). On the following day, the sections were PBS-rinsed, and then the sections were incubated for 1 h in biotinylated secondary goat anti-rabbit antibody (1:500). Following the PBS rinse, the sections were incubated for 1 h in ABC Elite diluted 1:1,000 in PBS. The sections were then washed for 30 min in PBS and submitted to a 10-min chromogen reaction with SG Kit or DAB. The sections were PBS-rinsed for a final time. Finally, the sections were rinsed (0.1 M phosphate buffer) and mounted; the slides were counterstained with neutral red, dehydrated, and cover-slipped.

Photomicrographs were obtained with a Leica DMLB microscope connected to a Leica DFC 300 digital camera or a Nikon A1R+ confocal microscope. The number of c-Fos+ cells was counted in the entire field of view at ×400 magnification in the AcbSh. Dual-labeled cells were labeled with both Fos and the second target, and only the dual-labeled neurons Fos+/other target+ are counted as “dual-labeled”. Cell-specific marker+ cells are the total number of cells expressing the marker (both single-labeled and dual-labeled combined). All cell counts were performed by an observer who was blind to the experimental treatment of each animal, and four to seven sections per brain were analyzed.

#### Experiment 4: effects of microinjection of 5-HT_7_ receptor agents into the AcbSh on the ability of a CS+ to enhance alcohol seeking or ability of a CS− to reduce alcohol seeking

**Figure 4:**
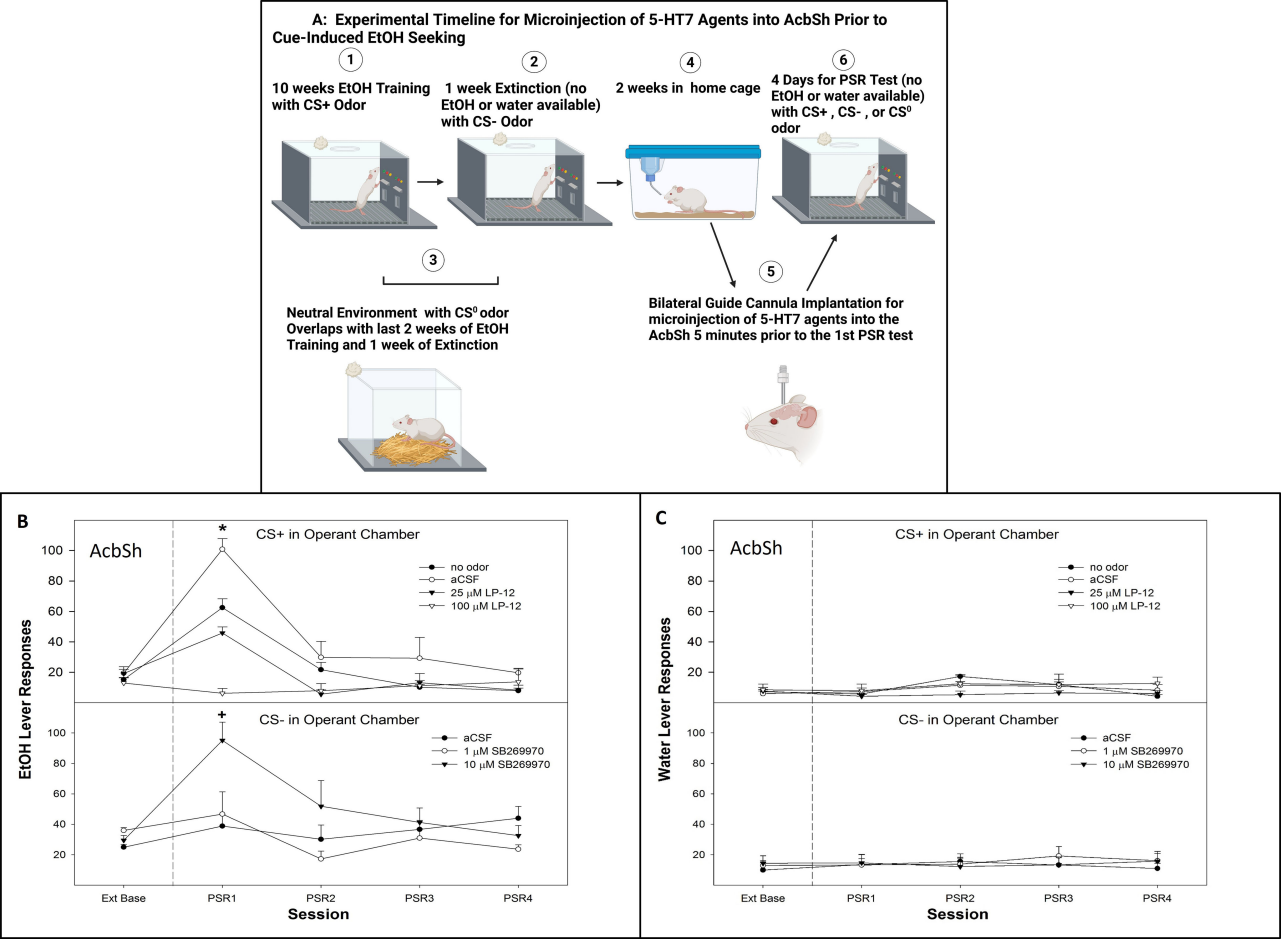
**(A)** Experimental timeline for the microinjection of 5-HT_7_ agonist or antagonist into AcbSh or core on cue-induced alcohol-seeking (created with BioRender.com). **(B)** Mean (± SEM) of responses on the lever previously associated with the delivery of alcohol during cue-induced alcohol-seeking behavior (PSR) in rats administered a 5-HT_7_ agonist (LP-12, top panel) microinjected into the AcbSh, while the CS+ was present in the operant chamber and a 5-HT_7_ antagonist (SB269970, bottom panel) microinjected into the AcbSh while the CS− was present in the operant chamber. **(C)** Mean (± SEM) of responses on the lever previously associated with the delivery of water during context-induced alcohol-seeking (PSR) testing in rats administered a 5HT_7_ agonist (LP-12, top panel) microinjected into the AcbSh, while the CS+ was present in the operant chamber and a 5HT_7_ antagonist (SB269970, bottom panel) was microinjected into the AcbSh while the CS− was present in the operant chamber. *indicates the following: (1) presentation of a CS+ enhanced alcohol-seeking behavior (CS+ > no odor), (2) alcohol-seeking behavior was observed in multiple groups (aCSF, no odor, and 25 µM LP-12 as well as greater responding compared to extinction baseline levels), and (3) microinjection of LP-12 into the AcbSh reduced the ability of a CS+ to enhance this alcohol-seeking behavior (25 µM LP-12 significantly less than aCSF controls, 100 µM LP-12 no difference from extinction baseline and significantly less than aCSF controls). + indicates that the 10 µM SB269970 expressed alcohol-seeking behavior (significant increase compared to the extinction baseline), and responding was significantly higher compared to the aCSF and 1 µM SB269970 groups which did not express alcohol-seeking behavior (no difference compared to the extinction baseline).

All rats were exposed to the standard conditioned cue protocol (CS+, CS−, and CS^0^; **Figure 4A** microinjection experimental timeline Created in BioRender. Hauser, S. (2025) https://BioRender.com/13ckrbc). Following an operant-conditioned training and during the second week of home cage, the rats were prepared for a bilateral stereotaxic implantation of a 22-gauge guide cannula (Plastic One, Roanoke, VA, USA) into the AcbSh (+1.7 mm AP, +2.4 mm lateral to the midline, and 7.5 mm ventral from the surface of the skull at a 10° angle) (47). While under isoflurane anesthesia (2%), the guide cannula was aimed 1.0 mm above the target region. The analgesics administered to the animals were bupivacaine hydrochloride 0.5% (1 mg/kg, administered once during surgery) and carprofen (10 mg/kg, administered subcutaneously, once during surgery and 2 days postoperatively). A 28-gauge stylet was placed into the guide cannula and extended 0.5 mm beyond the tip of the guide. After surgery, the rats were individually housed and allowed to recover for 7 days in their home cage. The animals were handled for at least 5 min daily beginning on the fourth recovery day and were habituated for two consecutive days to the handling procedures necessary for microinjections.

The rats were randomly assigned to groups (*n* = 5–6/group) that received (1) microinjections of aCSF, 25 or 100 µM LP-12 into the AcbSh 5 min prior to the first PSR test session and (2) microinjections of aCSF, 0.1, 1, or 10 µM SB269970 into the AcbSh 5 min prior to the first PSR test session. During the microinjection procedure, a 28-gauge injector was inserted to a depth of 1 mm beyond the end of the guide cannula in the AcbSh. The injector was connected to a Hamilton 25-μL syringe driven by a microinfusion syringe pump (Harvard Apparatus, Holliston, MA, USA). A total volume of 0.5 μL was administered over a 30-s period per side; the injector tip was left in place for an additional 30 s per side to allow for diffusion. An additional control group received aCSF microinjections into the AcbSh and was not exposed to the CS+ in the operant chamber (no odor control group).

To our knowledge, this is the first study to report the intracranial injection of LP–12 into a specific brain region. The LP-12 agonist has selectivity for 5-HT_7_ (Ki value 0.13 nM) over D_2,_ 5-HT_1A_ and 5-HT_2A_ receptors (Ki values 224, 60.9, and >1,000 nM, respectively). Previous studies have intracranially injected the 5-HT_7_ agonists LP-44 (Ki value 0.22 nM) and LP-211 (Ki value 0.58 nM) into targeted brain areas at doses in the millimolar range (51–53). Our lab chose to use doses for LP-12 in the micromolar range to further reduce the potential off-target behavioral effects. SB269970 (pKi 8.9) is the most commonly used 5-HT_7_ antagonist in research and has also been intracranially injected into specific brain regions at millimolar doses (51–53). This study opted for a dose–response approach in the micromolar range for SB269970.

#### Experiment 5: effects of microinjection of 5-HT_7_ receptor agents into the AcbSh on context-induced alcohol-seeking

**Figure 5:**
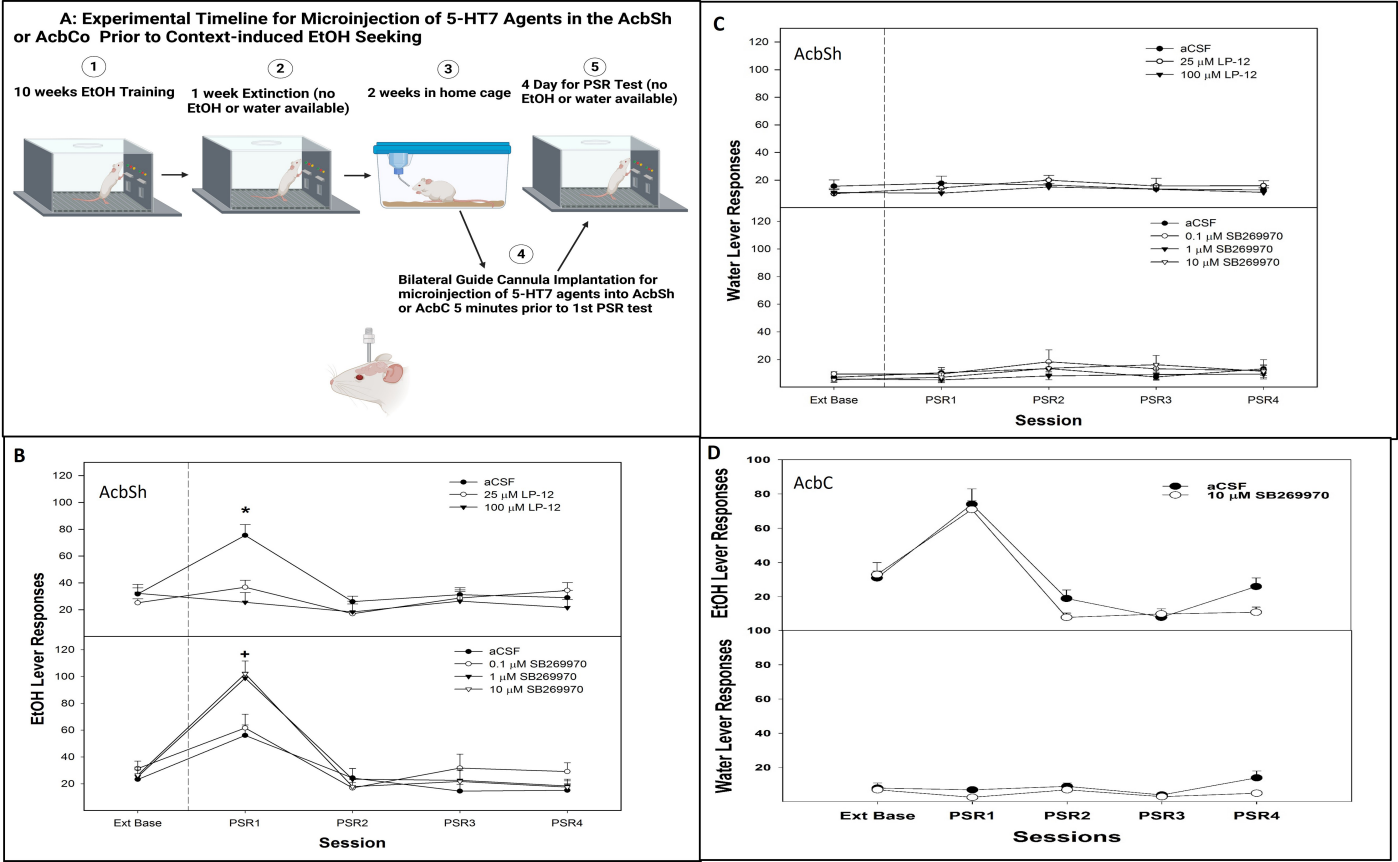
**(A)** Experimental timeline for microinjection of 5-HT_7_ agonist or antagonist into AcbSh or core on contextual alcohol-seeking (created with BioRender.com). **(B)** Mean (± SEM) of responses on the lever previously associated with the delivery of alcohol during context-induced alcohol-seeking behavior (PSR) in rats administered a 5-HT_7_ agonist (LP-12, top panel) or a 5-HT_7_ antagonist (SB269970, bottom panel) microinjected into the AcbSh. **(C)** Mean (± SEM) of responses on the lever previously associated with the delivery of water during context-induced alcohol-seeking (PSR) testing in rats administered a 5HT_7_ agonist (LP-12, top panel) or a 5HT_7_ antagonist (SB269970, bottom panel) via a microinjection into the AcbSh. **(D)** Mean (± SEM) of responses on the lever previously associated with the delivery of alcohol (top panel) and water (bottom panel) during context-induced alcohol-seeking (PSR) testing in rats administered a 5HT_7_ antagonist microinjected into the AcbC. *indicates that aCSF-treated rats expressed alcohol-seeking (increased PSR responding compared to baseline) and responded more than rats administered 25 or 100 µM LP-12 directly into the AcbSh (no evidence of alcohol-seeking behavior in these groups). +indicates that all groups expressed alcohol-seeking behavior (significant increase compared to extinction baseline) and that administration of 1 or 10 µM SB269970 directly into the AcbSh significantly augmented this alcohol-seeking behavior (significantly higher than aCSF or 0.1 µM SB269970 in lever responses).

The experimental protocol employed was the PSR model without odor cues being present at any time (14, 54, 55) to test 5-HT_7_ effects within Acb during context-induced alcohol seeking. After extinction training, stereotaxic implantation was performed 7 days into the home cage period (**Figure 5A**, microinjection experimental timeline; Created in BioRender. Hauser, S. (2025) https://BioRender.com/69t8njz). While under isoflurane anesthesia (2%), the rats were prepared for bilateral stereotaxic implantation of 22-gauge guide cannula (Plastic One, Roanoke, VA, USA) into the AcbSh (+1.7 mm AP, +2.4 mm lateral to the midline, and 7.5 mm ventral from the surface of the skull at a 10° angle) or AcbC (+1.7 mm AP, +2.7 mm lateral to the midline, and 6.5 mm ventral from the surface of the skull at a 10° angle); the guide cannula was aimed 1.0 mm above the target region. The analgesics administered to the animals during surgery were bupivacaine hydrochloride (1 to 2 mg/kg, administered once during surgery) and carprofen (10 mg/kg, administered subcutaneously, once during surgery and 2 days postoperatively). A 28-gauge stylet was placed into the guide cannula and extended 0.5 mm beyond the tip of the guide. After surgery, the rats were individually housed and allowed to recover for 7 days in their home cage. The animals were handled for at least 5 min daily beginning on the fourth recovery day and were habituated for two consecutive days to the handling procedures necessary for microinjections.

The rats were randomly assigned to groups (*n* = 5–8/group) that received (1) microinjections of aCSF, 25 or 100 µM LP-12 into the AcbSh 5 min prior to the first PSR test session, (2) microinjections of aCSF, 0.1, 1, or 10 µM SB269970 into the AcbSh 5 min prior to the first PSR test session, or (3) microinjections of aCSF or 10 µM SB269970 (*n* = 5–6/group) into the AcbC 5 min prior to the first PSR test session. During the microinjection procedure, a 28-gauge injector was inserted to a depth of 1 mm beyond the end of the guide cannula in the AcbSh or AcbC; the injector was connected to a 25-μL Hamilton syringe driven by a microinfusion syringe pump (Harvard Apparatus, Holliston, MA, USA). A total volume of 0.5 μL was administered over a 30-s period per side; the injector tip was left in place for an additional 30 s per side to allow for diffusion.

#### Experiment 6: the effects of systemic administration of 5-HT_7_ receptor agents on context-induced alcohol-seeking

**Figure 6:**
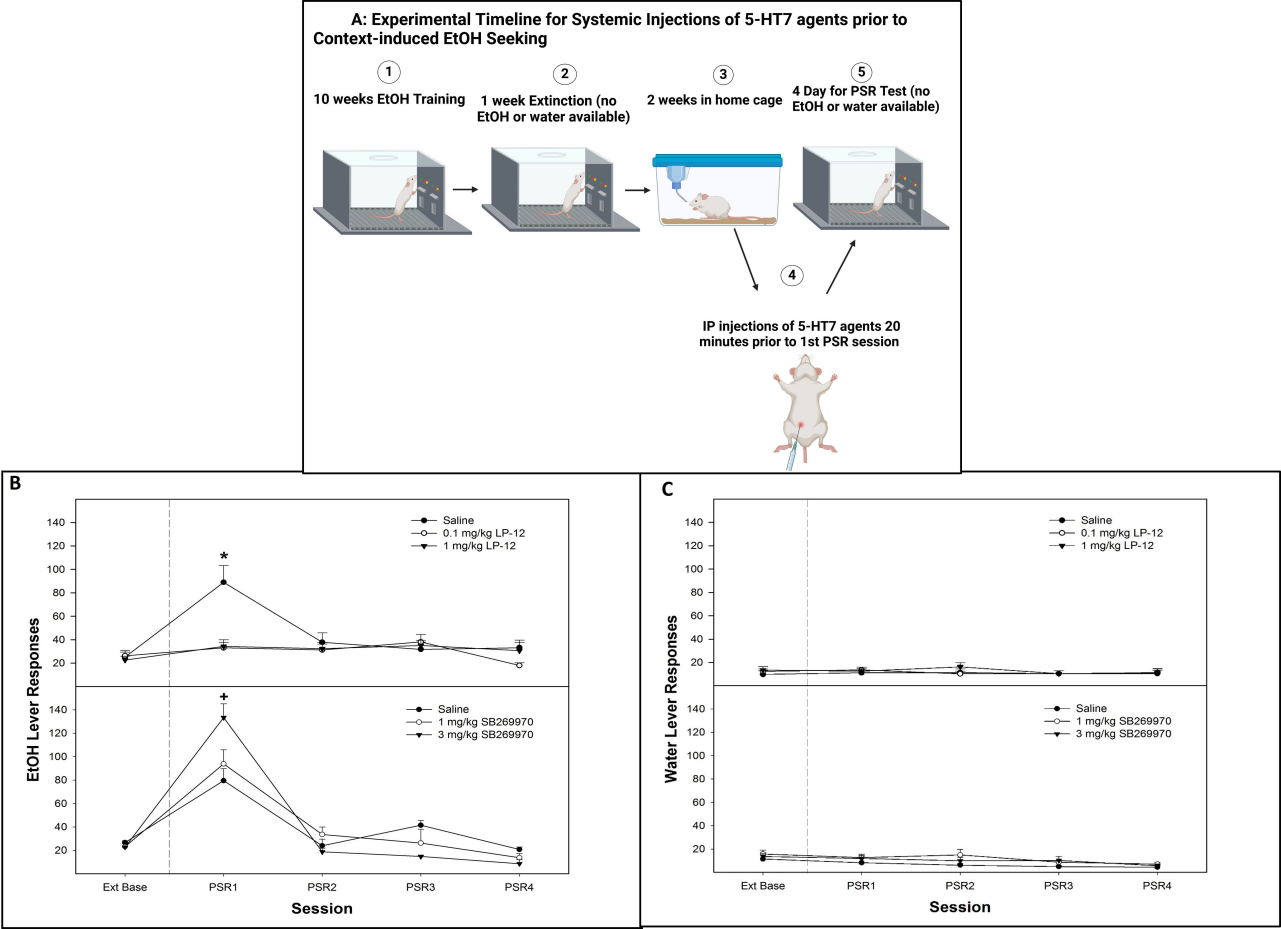
**(A)** Experimental timeline for the systemic administration of 5-HT_7_ agonist or antagonist on contextual alcohol-seeking (created with BioRender.com). **(B)** Mean (± SEM) of responses on the lever previously associated with the delivery of alcohol during context-induced alcohol-seeking (PSR) testing in rats systemically administered a 5-HT_7_ agonist (LP-12, top panel) or a 5-HT_7_ antagonist (SB269970, bottom panel). **(C)** Mean (± SEM) of responses on the lever previously associated with the delivery of water during context-induced alcohol-seeking (PSR) testing in rats systemically administered a 5HT_7_ agonist (LP-12, top panel) or a 5HT_7_ antagonist (SB269970, bottom panel). *indicates that saline-treated rats expressed alcohol-seeking behavior (increased responding compared to baseline) and responded more than rats administered 0.1 or 1 mg/kg LP-12 (no evidence of alcohol-seeking behavior in these groups). +indicates that all groups expressed alcohol-seeking (significant increase compared to extinction baseline) and that the administration of 3 mg/kg SB269970 significantly augmented the alcohol-seeking behavior (significantly higher than saline or 1 mg/kg SB269970 in lever responding).

The operant procedures are identical to those in experiment 5, where odor cues were not present (**Figure 6A** systemic injections experimental timeline, Created in BioRender. Hauser, S. (2025) https://BioRender.com/axt6m14). The rats were randomly assigned to groups (*n* = 6–8/group) that received saline, 0.1 or 1 mg/kg (i.p.) LP-12 (5-HT_7_ agonist; Tocris Bioscience, Minneapolis, MN, USA). At 20 min prior to the first PSR test session, the animals received injections of saline, 1 or 3 mg/kg (i.p.) SB269970 (5-HT_7_ antagonist; Tocris Bioscience, Minneapolis, MN, USA). The doses were selected based on the impulsivity study by Leo et al. (40), who used a 3-mg/kg dose of SB269970 and a 0.060-mg/kg dose for their 5-HT 1a/7 agonist 8-hydroxy-2(di-n-propylamino)tetralin.

### Statistical analyses

Overall operant responding (60 min) data were analyzed with a mixed factorial ANOVA with a between-factor of drug exposure and a repeated-measure of “session”. The baseline measure for the factor of “session” was the average number of responses on the alcohol lever for the last three extinction sessions (prior to the home-cage rest period). An analysis of cFos- and D1-immunoreactive cells was performed by one-way ANOVAs. For the microdialysis data, the values are presented as percent of baseline (average of the final three baseline samples). The initial analysis consisted of a conditioned cue × sample mixed analysis of variance (ANOVA). When significant effects (*p* < 0.05) were observed using the mixed ANOVAs, *post-hoc* Tukey’s *b*, or Student’s *t*-tests were utilized to determine differences between conditioned cues and across the time course of sample collection.

For all behavioral, neurochemical, and molecular biology studies, power analyses based on previous data were conducted to determine appropriate sample sizes. Power analyses were conducted using G*Power for Windows ^®^: within-group subject variance of the pilot group (25%); alpha level set at 0.05. The analyses indicated that *n*/group sizes of nine to ten/group/sex was necessary to achieve a power of.80.

## Results

### Experiment 1

The analysis revealed that for rats with probes located in the AcbSh (**Figure 1C**), there was a significant conditioned cue × sample interaction on the extracellular levels of DA (*F*_12, 246_ = 6.3; *p* < 0.001). In contrast, in rats with probes located in the AcbC (**Figure 1D**), there was no significant conditioned cue × sample interaction or an effect of conditioned cue or sample on DA levels (*p*-values > 0.32). Within the AcbSh, there was a significant effect of conditioned cue during the three odor presentation samples and the initial post-cue exposure sample (*F*_2,25_ values > 16.2; *p*-values < 0.001). *Post-hoc* comparisons indicated that the extracellular DA levels in the AcbSh in rats exposed to the CS− were significantly lower compared to the DA levels in rats exposed to CS^0^ and CS+ during the first and second odor exposure samples as well as being significantly lower than the baseline levels (paired *t*-tests, *p*-values < 0.05). In contrast, in rats exposed to CS+, the extracellular DA levels were increased during the second and third odor exposure samples and during the first post-cue exposure sample compared to the CS^0^ and CS− groups as well as being significantly greater than its baseline.

The control odor experiment (rats never exposed to alcohol but exposed to all conditioned cues) indicated that exposure to the different odors did not alter the DA levels in the AcbSh (**Figure 1E**), illustrating that odor exposure alone was not sufficient to produce alterations in extracellular DA levels.

### Experiment 2

The overall analysis revealed a significant conditioned cue × sample interaction on the extracellular levels of GLU in the AcbSh (**Figure 2A**, top panel; *F*_12, 186_ = 6.1; *p* < 0.001). There was a significant effect of conditioned cue during the three odor presentation samples and all post-cue exposure samples (*F*_2,25_ values >5.6; *p*-values < 0.007). *Post-hoc* comparisons indicated that the GLU levels in the AcbSh were significantly lower in rats exposed to CS− compared to the CS^0^ and CS+ groups for all post-exposure samples analyzed as well as baseline values.

The overall analysis revealed a significant conditioned cue × sample interaction on the extracellular levels of 5-HT in the AcbSh (**Figure 2B**, bottom panel; *F*_12, 174_ = 8.2; *p* < 0.001). There was a significant effect of conditioned cue only during the three odor presentation samples (*F*_2,27_ values > 5.6; *p*-values < 0.007). The *post-hoc* comparisons indicated that the 5-HT levels in the AcbSh were significantly lower in rats exposed to CS+ compared to the CS^0^ and CS− groups for the three odor exposed samples as well as being lower compared to baseline.

### Experiment 3

There was a significant effect of conditioned cue exposure on the total number of cFos+ (**Figure 3B**)-immunoreactive cells and dual-labeled cFos+/D1+ (**Figure 3C**) for cells within the AcbSh (*F*_2,13_ values > 10.84; *p*-values < 0.001). The *post-hoc* comparisons (Tukey’s b) indicated that rats exposed to CS+ had more total cFos+ and cFos+/D1+ cells in the AcbSh than the CS^0^ and CS− groups (which did not differ). However, there were significantly more cFos+ only cells (data not shown) in the AcbSh in rats exposed to CS+ than the other two groups (*F*_2,15_ = 15.5; *p* < 0.001). These data indicate that presentation of the CS+ activates D1 receptor containing neurons in the AcbSh as well as other cell types.

### Experiment 4

There was a significant experimental condition × session interaction following the micro-infusion of a 5-HT_7_ agonist LP-12 into the AcbSh of rats exposed to CS+ (**Figure 4B**, top panel; *F*_12, 164_ = 7.7; *p* < 0.001). Responding on the lever previously associated with the delivery of alcohol was only statistically significant between groups during the first PSR session (*F*_3, 16_ = 48.4; *p* < 0.001). The *post-hoc* comparisons indicated that the CS+ groups responded more than the other groups. The no odor and 25 µM LP-12 groups responded higher than baseline as well as more than the 100 µM LP-12 group.

The overall analysis revealed a significant dose × session interaction following the micro-infusion of a 5-HT_7_ antagonist SB269970 into the AcbSh of rats exposed to CS− (**Figure 4B**, bottom panel; *F*_8,64_ = 3.1; *p* = 0.005). The *post-hoc* comparisons indicated that rats microinjected with 10 µM SB269970 into the AcbSh responded more on the lever previously associated with alcohol delivery than rats administered aCSF or 1 µM SB269970. The latter two groups responded close to extinction baseline values. Administration of 5-HT_7_ agonist LP-12 (**Figure 4C**, top panel) or 5-HT_7_ antagonist SB269970 (**Figure 4C**, bottom panel) directly into the AcbSh did not influence water lever responding (all *p*-values > 0.05).

### Experiment 5

There was a significant dose × session interaction for the number of responses on the lever previously associated with the delivery of alcohol following the micro-infusion of a 5-HT_7_ receptor agonist LP-12 into the AcbSh (**Figure 5B**, top panel; *F*_8, 44_ = 11.3; *p* < 0.001). During the first PSR test session, there was a significant effect of dose (*F*_2,14_ = 6.8; *p* = 0.004). The *post-hoc* comparisons indicated that responding on the lever previously associated with the delivery of alcohol was significantly lower in rats administered 25 or 100 µM 5-HT_7_ receptor agonist LP-12 directly into the AcbSh compared to aCSF controls, which were elevated compared to baseline. The administration of LP-12 directly into the AcbSh did not influence water lever responding (all *p*-values > 0.71; **Figure 5C**, top panel).

There was a significant dose × session interaction for the number of responses on the lever previously associated with the delivery of alcohol following a micro-infusion of the 5-HT_7_ antagonist SB269970 into the AcbSh (**Figure 5B**, bottom panel: *F*_12, 92_ = 4.6; *p* < 0.001). During the first PSR test session, there was a significant effect of dose (*F*_3,23_ = 9.1; *p* < 0.001). The *post-hoc* comparisons indicated that responding on the lever previously associated with the delivery of alcohol was significantly higher in rats administered 1 or 10 µM SB269970 directly into the AcbSh compared to the aCSF or 0.1 µM SB269970 group. All groups displayed significantly higher responding compared to their respective extinction baselines (**Figure 5B**, bottom panel). The administration of SB269970 directly into the AcbSh did not influence water lever responding (all *p*-values > 0.56; **Figure 5C**, bottom panel).

The microinjection of 10 µM SB269970, the 5-HT_7_ antagonist, into the AcbC did not influence alcohol seeking (i.e., lacked an effect on operant lever responding, all *p*-values > 0.27). Specifically, rats administered aCSF and 10 µM SB269970 directly into the AcbC responded similarly during alcohol seeking (PSR) testing (**Figure 5D**, top panel), and 10 µM SB269970 produced a non-specific alteration in responding on the lever previously associated with water (**Figure 5D**, bottom panel).

### Experiment 6

There was a significant dose × session interaction for the number of responses on the lever previously associated with the delivery of alcohol (alcohol lever) following treatment with a 5-HT_7_ receptor agonist LP-12 (**Figure 6B**, top panel; *F*_8, 36_ = 6.4; *p* = 0.026). On the first PSR test session, there was a significant effect of dose (*F*_2,13_ = 4.5; *p* = 0.032). The *post-hoc* comparisons indicated that responding on the alcohol level was significantly lower in rats administered 0.1 or 1 mg/kg LP-12 compared to saline controls and did not differ from extinction baseline responding (**Figure 6B**, top panel). Responding on the alcohol lever by saline controls was significantly elevated compared to their extinction baseline. The administration of 5-HT_7_ receptor agonist LP-12 did not influence water lever responding (all *p*-values > 0.66; **Figure 6C**, top panel).

There was a significant dose × session interaction for the number of responses on the lever previously associated with the delivery of alcohol following the administration of the 5-HT_7_ antagonist SB269970 (**Figure 6B**, bottom panel; *F*_8, 40_ = 8.28; *p* = 0.002). During the first PSR test session, there was a significant effect of dose (*F*_2,12_ = 9.56; *p* = 0.005), with *post-hoc* comparisons indicating that the administration of the 3-mg/kg dose of SB269970 resulted in significantly higher alcohol lever responses than the saline and 1 mg/kg groups. Saline and 1 mg/kg dose were both elevated compared to extinction baseline (*p* < 0.05). The systemic administration of 5-HT_7_ antagonist SB269970 did not influence water lever responding (*p*-values > 0.36; **Figure 6C**, bottom panel).

## Discussion

The major findings of this study support previous research (5, 24, 25) in such a way that there are distinct neuro-circuits regulating the processing of excitatory and inhibitory conditioned cues. Previously, our lab demonstrated that a CS+ or CS− presented in a non-drug paired environment can enhance or decrease alcohol-seeking behavior, respectively. The cues also increase the neuronal activity in the AcbSh and AcbC of P rats (5). The current results extended these findings, indicating that presentation of a CS+ in a non-drug paired environment increases extracellular DA levels (**Figure 1B**) while decreasing 5-HT levels in the AcbSh (**Figure 2B**). In contrast, presentation of a CS− in a non-drug paired environment (removing both context and expression of drug-seeking behaviors) reduces the extracellular levels of both DA and GLU in the AcbSh (**Figures 1B**, **2A**).

Reduced DA neuronal activity (25, 56) has been associated with reward omission and predictive of a reinforcer being unavailable. Tobler et al. (25) reported that CS− exposure suppressed the majority of Acb DA neurons with only minor activations in the remaining cells tested, while CS+ activated all of the DA neurons that were tested, thus providing evidence that conditioned stimuli predicting reward access versus absence differentially activate DA neurons within the Acb. Moreover, Suto et al. (57) found that reward omission odors for cocaine attenuated the GLU levels, while availability odors increased the GLU levels in AcbSh and AcbC (57). These authors suggested that GLU-ergic inputs are involved in mediating reward expectancy in the Acb. GLU inputs from cortical/amygdala regions to Acb have been shown to regulate cue-induced alcohol-seeking behavior (58, 59), and the ablation of medial prefrontal inputs to the Acb reduces cue-induced alcohol-seeking behavior (59). Moreover, a CS− reduced GLU and DA release in the basolateral amygdala (BLA), while a CS+ did not alter the GLU levels but did increase DA release in the BLA (5). This suggests that a reduction in GLU activity in the BLA may be necessary for the CS− inhibition of alcohol-seeking behavior (5). It is interesting to note that in the current study, neither the CS+ nor the CS− had a significant effect on the extracellular DA levels in the AcbC (**Figure 1C**), suggesting a differential effect of conditioned cues (odors) on DA projections to the AcbSh vs. AcbC.

The current study findings also demonstrated that CS+ increased the activity in D1 receptor-containing cells in the AcbSh (**Figures 3B, C**). The Acb serves to integrate signaling from several mesocorticolimbic structures, thereby mediating goal-directed behavioral responses to obtain desired reinforcers (55, 60, 61). Activating D1 receptor-containing medium spiny neurons in the Acb evokes the performance of motivated behaviors (62, 63), and DA stimulation of D1 receptors in medium spiny neurons is a direct pathway to signal the expectation for a reinforcer/reward (64).

The reduction of symptoms associated with attention deficit/hyperactivity disorder, obsessive–compulsive disorder, and conduct disorder by the administration of methylphenidate (Ritalin) is associated with an upregulation of 5-HT_7_ receptors in the Acb (65). A subsequent study showed that the systemic administration of the 5-HT_7_ receptor antagonist SB269970 increased impulsive-like behavior (reduced behavioral “self-control” as measured by multiple behavioral screens), while a 5-HT_7_ agonist increased behavioral “self-control” (40). Moreover, an increase in 5htr7 mRNA expression in the AcbSh and AcbC was associated with reducing impulsive behaviors (40). Thus, within the Acb the 5-HT_7_ receptor is a positive modulator of behavioral inhibition. Moreover, human genetic studies have associated a *5htr7* polymorphism with a genetic predisposition to develop AUDs (66, 67). Interestingly, 5-HT_7_ receptors regulate neuroinflammation which has been implicated in AUDs, neurodegenerative disease, and other psychiatric conditions, such that 5-HT_7_ agonists have anti-inflammatory properties (68).

The current data indicate that activation of 5-HT_7_ receptors reduced cue-induced (**Figure 4B**, top panel) and context-induced (**Figures 5B**—top panel and **6B**—top panel) alcohol seeking, while inhibition of 5-HT_7_ receptors enhanced alcohol-seeking behavior (**Figures 4B**, **5B**, **6B**—bottom panels). Mechanistically, 5-HT_7_ receptors within the AcbSh are involved in this effect (**Figures 4**, **5**). In addition, the ability of 5-HT_7_ agents to bidirectionally modulate this behavior is indicative of direct modulation of the behavior by this receptor. The enhanced responding produced by the antagonist suggests a tonic inhibitory action of 5-HT at 5-HT_7_ that may limit the expression of cue- and/or context-induced alcohol-seeking behavior by P rats.

Manipulation of the 5-HT_7_ receptors in the AcbSh reversed the enhancement of alcohol-seeking behavior produced by a CS+ as well as attenuated the inhibition of alcohol-seeking behavior induced by the presentation of a CS− (**Figure 4**). Decreases in extracellular 5-HT levels within the AcbSh were observed in experiment 1 (**Figure 2B**). When the CS+ was present, it supports this notion and may indicate that presentation of an excitatory cue associated with past drug-taking produces a state of decreased behavioral inhibition. This may presumably occur from reduced 5-HT_7_ activity, which, in turn, results in lack of control over alcohol-seeking behavior. Overall, the data suggest that the 5-HT_7_ receptor is a potential therapeutic treatment targeting AUDs and possesses potential as a pharmacological intervention to decrease cue-induced relapse.

As previously mentioned, 5-HT_7_ receptors can also modulate DA firing activity VTA (39), and it has been suggested that the combination of 5-HT_7_ with D_1_/D_5_ modulation may improve behavioral flexibility, resulting in better adaptation to adverse situations (69, 70). The cFos+ only cells in the current study are not likely exclusive to D1 receptors, and it is possible that 5-HT_7_ receptors are also activated in the AcbSh. Therefore, further studies will need to be done to determine the interplay between 5-HT_7_ and D1 receptors in AcbSh on alcohol-seeking behaviors.

There are some limitations to the current study. Although we did not observe any lethargic behaviors in any of the animals treated with the 5-HT_7_ agents, future studies are needed to investigate the systemic and local effects of LP-12 and SB269970 in the AcbSh on locomotor activity behaviors and seeking behaviors for natural rewards (e.g., sucrose or saccharin). Additionally, clinical and preclinical studies have shown that craving levels and/or relapse tend to be higher in male rats compared to female rats (71); therefore, the involvement of 5-HT_7_ receptors in context- and cue-induced alcohol-seeking behaviors in male rats needs to be tested.

In conclusion, the data from this series of experiments indicate that excitatory and inhibitory conditioned cues exhibit unique and opposing effects on neuronal signaling in the AcbSh. One of the major factors precipitating relapse is the exposure to conditioned cues associated with past drug use. Thus, employment of inhibitory conditioned cues (CS−) in clinical settings may be a potential non-invasive therapeutic tool to decrease relapse rates as a behavioral treatment of addiction. Inhibitory cue exposure could be incorporated into mindfulness training, biofeedback, and other similar behavioral therapies to serve as an adjunct to conventional therapies. Furthermore, the efficacy of such treatments could potentially be further augmented by the pharmacological manipulation of the 5-HT system. The development of successful pharmacotherapeutics for the treatment of AUDs and drug craving may include the use of 5-HT_7_ modulators and their associated neuronal circuits regulating behavioral inhibition in the context of alcohol-seeking behavior.

## Data Availability

The raw data supporting the conclusions of this article will be made available by the authors, without undue reservation.
